# Supplementary material for: Understanding Grain Boundary Electrical Resistivity in Cu: The Effect of Boundary Structure
Source: ACS Nano. 2021 Oct 4;15(10):16607–15. doi: 10.1021/acsnano.1c06367 (PMC8552493; doi:10.1021/acsnano.1c06367)

Supplemental information

Understanding Grain Boundary Electrical Resistivity in Cu: The Effect of Boundary Structure

Hanna Bishara [(a)*], Subin Lee [(a)(b)], Tobias Brink [(a)], Matteo Ghidelli [(a)(c)], Gerhard Dehm [(a)*]

[(a)]Max-Planck-Institut für Eisenforschung GmbH, 40237 Düsseldorf, Germany

[(b)] Institute for Applied Materials (IAM), Karlsruhe Institute of Technology, 76344 Eggenstein-Leopoldshafen, Germany

[(c)] Laboratoire des Sciences des Procédés et des Matériaux (LSPM), CNRS, Université Sorbonne Paris Nord, 93430 Villetaneuse, France

* Corresponding author: h.bishara@mpie.de ; g.dehm@mpie.de

Table S1:

List of inspected [111] tilt grain boundary (GB) segments for which GB resistivity was measured. In case of symmetric GBs only one of the GB normals is given since the GB normal of the other grain is identical.

GB type	Misorientation (°)	GB plane normals	GB Resistivity (10^{-12} Ωcm^2)
Low angle GBs	7	[1 -4 3] ; [1 -8 7]	0 ± 2
	13.8	[1 -3 2] ; [1 -10 9]	20.1 ± 1.5
	14	[-10 -1 11] ; [-2 -1 3]	19.7 ± 1.2
	16	[1 -4 3] ; [-2 1]	22.9 ± 1.7
	16.6	[3 -5 2] ; [7 -8 1]	24.8 ± 2.8
Σ 21a	20	[3 -1 -2]	17.3 ± 3.5
Σ 7	42.3	[3 2 -5] ; [3 -1 -2]	15 ± 1.7
	42.4	[2 -5 3] ; [10 -9 -1]	16.1 ± 2
	42.7	[3 2 -5] ; [3 -1 -2]	16 ± 2
Σ 19b	43.7	[3 -1 -4] ; [2 -1 -1]	10.4 ± 1.2
	44.4	[1 0 -1] ; [4 -3 -1]	10.2 ± 2.7
	45.5	[2 -3 -1] ; [9 -5 -4]	9.9 ±2
	45.6	[2 3 -5]	12.8 ± 1.8
	47.5	[1 -8 7]	14.5 ± 4.5
Σ 37c	51.3	[5 -1 4] ; [2 3 -5]	7.3 ± 2
	52.1	[3 1 -4] ; [5 -3 -2]	6.7 ± 1.5
	53.3	[1 3 -4] ; [-2 3 -1]	5.4 ± 1.4
Σ 3	58	[1 -1 0]	1.13 ± 0.26
	58.5	[1 -1 0]	1.25 ± 0.17
	59.5	[1 -1 0]	1.24 ± 0.1
	60	[1 -1 0]	1.18 ± 0.18

Figure S1:

Snapshots of the GB phases discovered using MD simulations viewed from the ⟨111⟩ direction. The two possible symmetric variants are shown in the left and right column, respectively. For Σ21a {123} and Σ19b {178}, two different phases can occur. Atoms inside the GB are highlighted in red to emphasize the structural motifs and guide the eye. They do not correspond to a structural analysis. The black bars indicate the GB unit cell. The scale bar applies to all images.

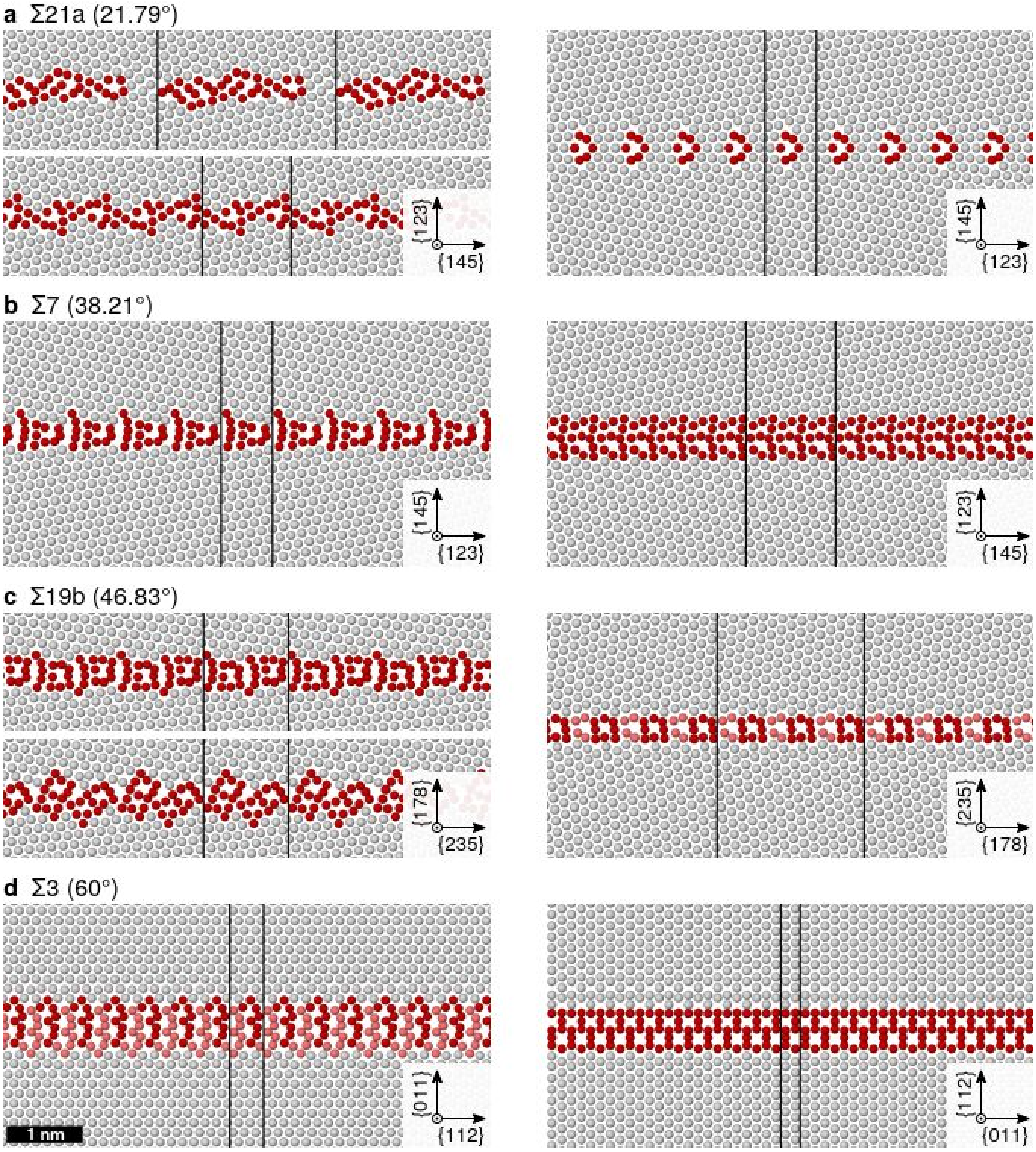

Supplement: Supplementary file 1 — nn1c06367_si_001.pdf [file nn1c06367_si_001.pdf]
